# Idiopathic membranous nephropathy in pediatric patients: presentation, response to therapy, and long-term outcome

**DOI:** 10.1186/1471-2369-8-11

**Published:** 2007-08-06

**Authors:** Ashton Chen, Rachel Frank, Suzanne Vento, Virginia Crosby, Manju Chandra, Bernard Gauthier, Elsa Valderrama, Howard Trachtman

**Affiliations:** 1 Department of Pediatrics, Division of Nephrology, Schneider Children's Hospital of North Shore-Long Island Jewish Health System, New Hyde Park, NY 11040-1432, USA; 2 Department of Pathology, Schneider Children's Hospital of North Shore-Long Island Jewish Health System, New Hyde Park, NY 11040-1432, USA

## Abstract

**Background:**

Idiopathic membranous nephropathy (IMN) is one of the most common causes of primary nephrotic syndrome in adults. However, it is a relatively rare entity in the pediatric population and there is a paucity of data about the incidence, prognosis, and optimal treatment of IMN in children and adolescents. We conducted this study to evaluate pediatric patients with IMN in order to clarify the presentation, response to therapy, and clinical outcome.

**Methods:**

A retrospective chart review was performed on patients identified with biopsy-proven IMN between 1988–2005. Patients with systemic lupus erythematosus or hepatitis-related lesions were excluded. The following data were tabulated: age, gender, ethnicity, presenting clinical and laboratory findings, proteinuria in a first morning urine specimen, estimated glomerular filtration rate (GFR_e_), histopathology, type and duration of treatment, and clinical status at final evaluation.

**Results:**

13 cases of IMN were identified out of 460 renal biopsies performed for evaluation of primary kidney disease during the study interval. Mean age was 9.6 ± 4.6, gender 6 M:7 F, ethnicity 8 W:2 B:3 H. At the initial visit hematuria was present in 9 patients, edema in 5, nephrotic-range proteinuria in 5, and hypertension in 3. Mean urinary protein:creatinine ratio 3.3 ± 2.5 and all patients had a normal GFR_e_. Classic glomerular findings of IMN were seen in all renal specimens, with concomitant interstitial changes in 2 cases. Treatment included an angiotensin converting enzyme inhibitor or angiotensin receptor blocker in 11 cases. Most patients were also given immunosuppressive medications – prednisone in 10, a calcineurin inhibitor in 5, and mycophenolate mofetil or azathioprine in 3 patients. At the last follow-up, 42 ± 35 months after the diagnostic biopsy, 7 children were hypertensive and the urine protein:creatinine ratio was 2.3 ± 3.1. The mean GFR_e _was 127 ± 57 mL/min/m^2^. Three patients had Chronic Kidney Disease Stage 3, all of whom were also hypertensive.

**Conclusion:**

IMN is a rare but serious glomerulopathy in pediatrics. We estimate that it accounts for approximately 3% of renal biopsies. Long-term prognosis is guarded because approximately 50% of patients may have evidence of progressive kidney disease.

## Background

Although idiopathic membranous nephropathy (IMN) is one of the most common etiologies of nephrotic syndrome in adults, it is an uncommon entity in the pediatric population [1]. When it is identified in childhood, membranous nephropathy is often a result of co-morbid illnesses, such as SLE, hepatitis B or C infection, or administration of various medications [1]. After excluding these secondary causes, IMN is extremely rare. There is a paucity of data about the prognosis and optimal treatment of IMN in children and adolescents. Therefore, we conducted this single-site, retrospective review of IMN in children and adolescents to clarify these unresolved issues.

## Methods

A retrospective chart review was performed on all patients identified with biopsy-proven idiopathic membranous nephropathy (IMN) at Schneider Children's Hospital between 1988–2005. Patients were identified by scanning a database maintained in the Division of Nephrology. They were eligible for inclusion in this study if they had histopathological findings consistent with IMN. Patients with SLE, hepatitis, or any other secondary causes were excluded based on history and negative laboratory studies. Pathology records were reviewed to determine the number of renal biopsies done during the study period for the evaluation of potential primary kidney disease.

The following data at initial presentation were recorded: age, gender, ethnicity, clinical and laboratory findings, estimated glomerular filtration rate (GFR_e_), and histopathology based on renal biopsy. Ethnicity was classified in accordance with NIH guidelines. Clinical findings noted at presentation included height, weight, and blood pressure, and presence or absence of edema. Laboratory findings included BUN, serum creatinine, albumin, and cholesterol concentrations, hematuria (defined as positive if the reading was more than trace), and urine protein:creatinine ratio in a first morning specimen. Nephrotic-range proteinuria was defined as a ratio >2. GFR_e _(mL/min/1.73 m^2^) was calculated using the age-appropriate Schwartz formula [2]. Chronic kidney disease (CKD) was classified according to the NKF/DOQI guidelines [3]. Blood pressures were evaluated using the 2004 National High Blood Pressure Education Program Working Group guidelines, based on gender, age, and height percentiles [4]. The summaries of the histopathology findings were obtained from the pathological report. The presence or absence of interstitial disease, the predominant immunoglobulin deposited in subepithelial space, and presence of glomerular sclerosis were noted.

For each patient, the type of medication and the total duration of therapy were recorded. At the final follow-up visit, the same data set extracted at the time of the disease presentation was recorded.

Information was gathered using a pre-approved form and data were deidentified and recorded by study number in accordance with HIPAA guidelines. This retrospective chart review was approved by the Institutional Review Board of the North Shore-Long Island Jewish Health System.

Data are presented as mean ± SD. The difference between groups was evaluated using the Student t-test. Differences in outcome between groups defined by the presence or absence of specific clinical findings at presentation were assessed using the Fisher exact test. Results were considered statistically significant if the P value was less than 0.05.

## Results

Over the 17-year study period, 13 patients with biopsy-proven IMN were identified (Table 1). The mean age at presentation was 9.6 ± 4.6 years (range: 4–17 years), and 9 patients were ≤ 10 years old. Six patients were male. Eight patients were White, 2 patients were Black, and 3 were of Hispanic ethnicity. When they were first evaluated, 9 patients had microscopic hematuria, 5 had peripheral edema, 5 had nephrotic-range proteinuria, and 3 patients were hypertensive. Less common symptoms were abdominal pain noted in 2 patients and nocturnal enuresis in 1 patient. At presentation, all patients had a normal serum creatinine concentration and the GFR_e _was 155 ± 34 mL/min/1.73 m^2^. No patient had a reduced GFR_e_, i.e., <90 mL/min/1.73 m^2^. Six patients had hypoalbuminemia and all 13 patients had hypercholesterolemia. The urinary protein:creatinine ratio at the initial assessment was 3.3 ± 2.5. (Table 1)

**Table 1:** IMN in pediatric patients: clinical and laboratory data (N = 13)

	**Initial Visit N (%)**	**Final Visit n (%)**
**Mean Age**	9.6 ± 4.6	--
**Gender**	6 M:7 F	--
**Ethnicity**	8 W:2 B:3 H	--
**Hematuria**	9 (69%)	6 (46%)
**Peripheral Edema**	5 (38%)	0
**Proteinuria (protein:creatinine ratio)**	3.3 ± 2.5	2.3 ± 3.1
**Nephrotic-range Proteinuria**	5 (38%)	3 (23%)
**Hypoalbuminemia**	6 (46%)	5 (38%)
**Hypertension**	3 (23%)	7 (54%)
**Hypercholesterolemia**	10 (77%)	5 (38%)
**GFR**_e_**mL/min/m**^2^	155 ± 34	127 ± 57
**CKD Stage 3–5**	0	3 (23%)

Abbreviations: CKD, Chronic kidney disease; GFR_e_, Estimated glomerular filtration rate

The mean interval between referral to pediatric nephrology and performance of a kidney biopsy was 1.7 ± 1.5 months. Characteristic histopathological findings of IMN were seen in glomeruli in all specimens. Three patients were identified as having glomerulosclerosis, 2 of whom also had tubulointerstitial disease. Precise staging was done in only 4 patients with 3 patients classified a Stage II and 1 as Stage III. Eleven patients had subepithelial deposits on biopsy, 8 had intramembranous changes, and 2 had resorption of deposits. Diffuse or localized podocyte fusion was noted in 11 patients. The predominant immunoglobulin in the subepithelial or intramembranous deposits was IgG in all 12 patients for whom this information was recorded. Of these, 2 had combined IgG and IgM and 1 had IgG and IgA deposition.

**Figure 1:**
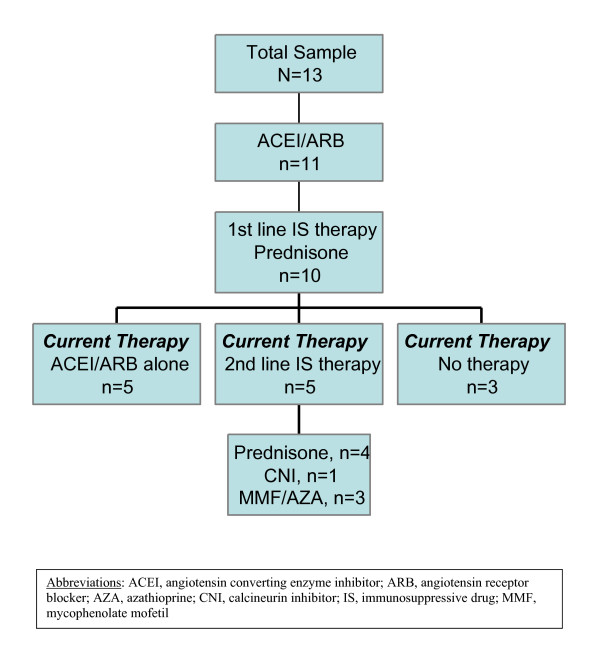
Summary of treatment provided to the patients with IMN.

Eleven out of the 13 patients received an angiotensin converting enzyme inhibitor (ACEI) or angiotensin receptor blocker (ARB) for 20 ± 21 months. Ten patients were also given immunosuppressive therapy with prednisone as the first-line therapy. The criteria for the use of prednisone were persistence of proteinuria after implementation of treatment with ACEI and/or ARB. The initial dosage of prednisone was 60 mg/m^2^/day for 4–6 weeks followed by 40 mg/m^2 ^every other day. The duration of prednisone treatment was 16 ± 19 months and the medication was discontinued without tapering. Six of the patients went on to receive one or more second-line drugs: cyclosporine (n = 3), tacrolimus (n = 2), and/or mycophenolate mofetil/azathioprine (n = 3). There were no consistent criteria or clinical guidelines for the administration of these agents during the study period. The cumulative duration of secondary immunosuppressive therapy was cyclosporine 22 ± 12 months, tacrolimus 11 ± 2 months, and mycophenolate mofetil/azathioprine 15 ± 16 months. Proteinuria fluctuated over the course of treatment without clear-cut relapses or remissions. Figure 1 summarizes the overall changes in treatment during the follow-up period.

At last follow-up, 42 ± 35 months after the diagnostic biopsy, none of the patients had peripheral edema, 6 had hematuria, 5 had hypoalbuminemia, and 4 were hypertensive. In the complete cohort the GFR_e _was 127 ± 57 ml/min/1.73 m^2 ^and the urine protein:creatinine ratio was 2.3 ± 3.1 (Table 1). Although these values were lower than original levels, the changes were not significant (P > 0.15). 3 patients had an elevated serum creatinine concentration with a GFR_e _of 37 ± 9 mL/min/1.73 m^2^(CKD Stage 3), compared with the remaining patients who had a GFR_e _157 ± 22 mL/min/1.73 m^2^.

None of the following clinical features at initial presentation – hematuria, nephrotic-range proteinuria, or hypertension – had any predictive value in determining which patients would develop hypertension or compromised GFR during the follow-up period, reflecting the small sample size. Nonetheless, it is worth noting that all 3 patients with hypertension at the onset of the disease had CKD and remained hypertensive at the last follow-up visit.

## Discussion

In this single center retrospective review, we describe 13 cases of IMN identified over a 17 year time period. Based on a total of 715 renal biopsies done during the study period, we estimate that the likelihood of a patient who undergoes this procedure for evaluation of glomerular disease having IMN is approximately 2%. Although IMN is a rare disease in the pediatric population, it can occur in the first decade of life as illustrated by our finding that 9 out of the 13 cases were documented in children less than 10 years of age. Males and females are equally affected and the disease occurred in all ethnic groups. The predominance of white patients (62%), compared to Blacks (15%) and Hispanics (23%), reflects the demographics of our institution. Nonetheless, the gender and ethnic distribution in our series is similar to other reports of the disease in children and adolescents [5].

The patients with IMN presented with varied signs and symptoms, most commonly hematuria (75%), proteinuria (50%), and edema (38%), consistent with other publications [5]. Patients may have hypoalbuminemia but most have hypercholesterolemia. The reason for the dissociation between the changes in serum albumin and cholesterol is unclear. All patients had normal renal function, but nearly a quarter had hypertension at the time of diagnosis.

In our study, histopathological staging of IMN in the renal biopsies was not standard procedure [6]. There are several reports in the literature indicating that in adults this procedure is helpful in predicting prognosis and the response to drug therapy [7,8]. In children, Ramirez, et al [9] reported an increased likelihood of progression to renal insufficiency with Stage III and IV on initial biopsy compared to Stage I or II lesions. However, Latham P et al [10] described no correlation between initial histological staging and clinical outcome. Obana et al [11] recently reported segmental *versus *diffuse glomerular involvement and proposed that these patterns may represent two disease entities or subcategories of IMN. However, staging was not evaluated as a prognostic tool in this article.

There is a great deal of discussion about the nature and duration of treatment for IMN in adult patients without a clear consensus on the most appropriate therapeutic choice [12]. Prednisone is the standard first-line immunosuppressive therapy for IMN in pediatric patients and steroid-resistant disease is the usual indication to initiate treatment with another immunosuppressive agent. It is difficult to assess the efficacy of the second-line immunosuppressive drugs prescribed in our study because these medications were only initiated after the patient was refractory to prednisone. Thus, bias may be introduced based on administration to those who were refractory to steroids and who may have had more severe kidney disease. In addition, we are unable to compare efficacy between mycophenolate mofetil, azathioprine, cyclosporine, and tacrolimus because the small number of patients in our study precludes such an analysis. Moreover, some patients received two or more second-line agents. This reflects the infrequent occurrence of IMN in pediatrics and the single center nature of this report. A collaborative randomized clinical trial will almost certainly be required to evaluate efficacy of immunosuppressive medications in children and adolescents with IMN.

ACEI and/or ARB therapy are renoprotective in adult patients with glomerular disease. We are unable to compare outcomes in patients receiving ACEI or ARB therapy *versus *those who did not, because only two patients were not treated with one of these antihypertensive agents. If these drugs are used alone, they may be insufficient to adequately control blood pressure or retard disease in patients with IMN. Thus, other antihypertensive medications should be prescribed to control blood pressure in addition to maximum tolerated antiproteinuric doses of ACEI and/or ARB therapy, given the increased incidence of hypertension that we observed during the follow-up period.

At the time of the final assessment, the mean GFR_e _decreased from initial presentation, with 3 patients (23%) having CKD Stage 3, described as moderate decrease in GFR_e _[3]. These three patients were also hypertensive and were the same three patients who presented with hypertension at the onset of their disease. Moreover, 4 other patients developed hypertension with maintenance of normal GFR_e_. Thus, there was an increased proportion of patients with hypertension from 23% at initial presentation to 54% at last the follow-up visit. In spite of immunosuppressive therapy, involving more than one class of drug in some patients, IMN can be a progressive disease in children and adolescents.

In the last three decades, there have been 7 other reports of IMN in children that contained at least 10 patients. (Table 2) Most estimate an occurrence rate of IMN between 1.0–6.7% of all renal biopsies performed at their institution [9-11,13-16], which is consistent with our observations. They all confirm that the disease can occur in children <10 years old. The gender distribution ranges from an even split as in our series [9,15,16] to a male predominance [10,11,13,14]. There is wide variability in the percentage of patients presenting with nephrotic-range proteinuria, as well as the degree of long-term renal morbidity. Our outcome data are most similar to Latham et al [10] and Trainin et al [15] who reported that 21–29% of patients manifested renal insufficiency at the last follow-up. Most studies in adult patients with IMN suggest that at least a quarter progress to CKD [17]. The presence of hypertension alone at last follow-up in 4 out of our 13 patients, an observation not routinely reported in the pediatric studies, raises additional concerns about the prognosis in those patients. It is worth noting that in Latham's study [10], children with IMN who had hypertension at the onset of their disease had a lower likelihood of entering remission. The marked variability in outcome in pediatric patients with IMN may depend on differences in ethnicity, biopsy practice patterns, and genetic factors. This argues in favor of exercising caution when discussing prognosis with individual pediatric patients found to have IMN.

**Table 2:** Prior reports of IMN in pediatric patients: a comparative review

**Author, Year**	**N**	**Follow-up Period (yr)**	**Mean Age at Presentation (yr)**	**Age Range**	**M:F**	**Nephrotic at Presentation (%)**	**↓ GFR at last F/U (%)**	**HTN at last F/U (%)**	**Estimated Occurrence Rate (%)**
Habib et al., 1973	50	1–10	NA	8 mo-14 yr	38:12	62	10	NA	3.7
Trainin et al., 1976	14	5.5	9	2–15 yr	6:8	79	29	NA	6.7
Ramirez et al., 1982	22	4.7	12	11 mo-19.9 yr	11:11	77	37	NA	5.7
Latham et al., 1982	14	6.4	10.5	3.5–14	9:5	79	21	NA	1.3
Tsukahara et al., 1993	12	5.9	7.7	2.9–15.8 yr	8:4	25	0	0	3
Obana et al., 2006	38	7.5 (SMGN); 12.4 (GMGN)	7.6	1.5–16 yr	25:13	24	0	5%	2.4
Lee et al., 2006	19	28.5	9.5	1.7–14.9	9:10	89.5	15	NA	1
*Current study*	*13*	*3.5*	*9.6*	*4–17 yr*	*6:7*	*38*	*23*	*54%*	*2.8*

Abbreviations: GMGN, global membranous glomerulonephritis; HTN, hypertension; NA, not available; SMGN, segmental membranous glomerulonephritis

## Conclusion

In summary, (1) IMN is a rare disease in childhood accounting for approximately 3% of all biopsies done in pediatric patients; (2) The long-term prognosis of IMN should be considered guarded because nearly 50% of patients will have CKD and/or hypertension after a 4 year follow-up period; and (3) Although prednisone is the current mainstay of immunosuppressive therapy, systematic evaluation of the efficacy of other immunosuppressive drugs in children and adolescents with IMN is urgently required building on the experience of randomized clinical trials performed in adult patients (18,19,20,21,22). The rarity of IMN in pediatrics limits its study and the challenge of identifying optimal treatment will mandate a collaborative approach.

## Abbreviations

ACEI: angiotensin converting enzyme inhibitor

ARB: angiotensin receptor blocker

CKD: chronic kidney disease

GFRe: estimated glomerular filtration rate

HTN: hypertension

IMN: idiopathic membranous nephropathy

SLE: systemic lupus erythematosus

## Competing interests

The author(s) declare that they have no competing interests.

## Authors' contributions

AC, RF, SV, MC, BG, and HT designed the retrospective data collection form. AC, RF, SV, and VC collected the data. AC, HT, MC and BG analyzed the data. AC, HT and BG wrote the manuscript. EV reviewed the renal biopsies and summarized the pathology data.

## Pre-publication history

The pre-publication history for this paper can be accessed here:


